# Education—General and Technical

**Published:** 1899-02-11

**Authors:** 


### The Hospital

A Journal of

The Medical Sciences and Hospital Administration.

Vol. XXV.—No. 646.] EDITED BY SIR HENRY BURDETT, K.C.B., AND SOLOMON C. SMITH, M.D., M.R.C.P. [Feb. 11, 1899.

### Education—General and Technical.

The thoughtful and suggestive address delivered by Mr. Balfour, at the opening of the new Hall in connection with the Battersea Polytechnic, contained much that should be of interest to all classes of the community, and especially to all who are in any way responsible for the bodily and mental training of the coming generation, to whose brains and hands the destinies of the British Empire will be committed when those who now control them have passed away. In dealing with the various senses which are attached to the word "education," and in pointing out the confusion which is apt to occur between them, Mr. Balfour might have remembered the comprehensive definition by Paley which includes them all, and, according to which, "education" comprises "every preparation which is made in our youth for the sequel of our lives." Perhaps the most remarkable passage of the address, taken singly, was that in which the speaker contrasted the relative positions of scientific research and industrial improvement in the time of Newton and in our own day; pointing out, in effect, that science and industrial improvement, although always moving upon converging lines, were still, in the time of Newton, too far apart to exert any immediate influence upon each other, while now, in the time of Pasteur and of Lord Kelvin, they have approached so closely that every advance on the one is immediately attended by a corresponding advance on the other. But it does not follow, and in the order of the greatest progress it is never likely to follow, that the advances upon the two lines will be directed by the same individual. The philosopher described by Tyndall, who replied to the importunities of a manufacturer that "he had no time to waste in making money," must continue to be represented if the advancement of knowledge which has marked the last half century is to be carried on throughout the next.

The general convergence of discovery and application, conspicuously manifest as it is in many departments of knowledge, is perhaps least of all conspicuous in its influence upon education. The principles which govern the useful employment of brain and fingers fall entirely within the domain of physiology, while the efforts at present made to cultivate mental capacity, and to confer manual dexterity, are almost absolutely guided not only by empiricism, but by an empiricism in which all the successes are claimed by the teacher, while all the failures are laid to the account of the pupil. The science which may ultimately teach us to make the best of every human organism is at present, even if it can be said to exist, only in the earliest stage of its infancy; but it is not extravagant to look forward to a time at which it may overshadow and surpass all other branches of knowledge. The waste of humanity in our own day is probably less than at any former period of the world's history, but none the less it is the greatest and most deplorable of all the forms of waste which we see around us. A large proportion of the available capacity of the race is thrown away by what must still be called the accidental entanglement of square pegs in round holes, and by the absence of any of the forms of knowledge by which it is conceivable that the shapes and dimensions alike of pegs and of holes will one day be accurately determined. The researches into the minute structure of nerve tissue, and into the changes wrought in it by circumstances or by employment, which are rendered possible by improved methods of preparation and by improved methods of investigation, the researches of such workers as Professor Sully into the development of ideation in the young, the labours of physicians in medical charge of great schools, are all leading us in the direction of real knowledge with respect to the values and uses of educational processes, and may perhaps be said to bear, to the education of the future, a relation like that of the first spark which Faraday obtained from a magnet to the electricity which now affords light to innumerable homes. In the meanwhile, of course, we must seek to employ to the best advantage such resources as the actual state of knowledge places at our disposal; and, among these, Mr. Balfour is unquestionably right in assigning a high value to the "polytechnic" system which has come into existence within the last twenty years, and which now affords such great facilities for the acquisition of many kinds of useful knowledge. Not the least of its merits will be its provision for social as well as for technical education, for the most conspicuous defect in the young people of the present day of the class for whom "polytechnics" are chiefly intended is a general want of that true politeness which arises from habitual consideration for the wishes and feelings of others. The "scorcher," or the young man with a filthy pipe in his mouth, who puffs tobacco smoke into the faces of women waiting their turn at the ticket office of a railway station, is a character which the social side of a polytechnic may be expected to do something to reform.

